# Inherited variants in *XRCC2* and the risk of breast cancer

**DOI:** 10.1007/s10549-019-05415-5

**Published:** 2019-08-28

**Authors:** Wojciech Kluźniak, Dominika Wokołorczyk, Bogna Rusak, Tomasz Huzarski, Jacek Gronwald, Klaudia Stempa, Helena Rudnicka, Aniruddh Kashyap, Tadeusz Dębniak, Anna Jakubowska, Marcin Lener, Marek Szwiec, Joanna Tomiczek-Szwiec, Joanna Jarkiewicz-Tretyn, Magdalena Cechowska, Paweł Domagała, Agata Szymiczek, Maryam Bagherzadeh, Jan Lubiński, Steven A. Narod, Mohammad R. Akbari, Cezary Cybulski, the Polish Hereditary Breast Cancer Consortium, M. Bębenek, D. Godlewski, S. Gozdecka-Grodecka, S. Goźdź, O. Haus, H. Janiszewska, M. Jasiówka, E. Kilar, R. Kordek, B. Kozak-Klonowska, G. Książkiewicz, A. Mackiewicz, E. Marczak, J. Mituś, Z. Morawiec, S. Niepsuj, R. Sibilski, M. Siołek, J. Sir, D. Surdyka, A. Synowiec, C. Szczylik, R. Uciński, B. Waśko, R. Wiśniowski, T. Byrski, B. Górski

**Affiliations:** 1 Department of Genetics and Pathology, International Hereditary Cancer Center, Pomeranian Medical University in Szczecin, Unii Lubelskiej 1, 71-252 Szczecin, Poland; 2 Department of Clinical Genetics and Pathology, University of Zielona Góra, Zielona Góra, Poland; 3 Independent Laboratory of Molecular Biology and Genetic Diagnostics, Pomeranian Medical University in Szczecin, Szczecin, Poland; 4 Department of Surgery and Oncology, University of Zielona Góra, Zielona Góra, Poland; 5 Department of Clinical Oncology, University of Zielona Góra, Zielona Góra, Poland; 6 Faculty of Natural Sciences and Technology, University of Opole, Opole, Poland; 7 Department of Oncological Gynecology, Oncology Center in Opole, Opole, Poland; 8 Cancer Genetics Laboratory, Genetic Outpatients Clinic in Toruń, Toruń, Poland; 9 Department of Pathology, Pomeranian Medical University in Szczecin, Szczecin, Poland; 10 Women’s College Research Institute, Women’s College Hospital, University of Toronto, Toronto, Canada; 11 Dalla Lana School of Public Health, University of Toronto, Toronto, Canada

**Keywords:** *XRCC2*, Mutation, Breast cancer, Hereditary

## Abstract

**Background:**

*XRCC2* participates in homologous recombination and in DNA repair. *XRCC2* has been reported to be a breast cancer susceptibility gene and is now included in several breast cancer susceptibility gene panels.

**Methods:**

We sequenced *XRCC2* in 617 Polish women with familial breast cancer and found a founder mutation. We then genotyped 12,617 women with breast cancer and 4599 controls for the *XRCC2* founder mutation.

**Results:**

We identified a recurrent truncating mutation of *XRCC2* (c.96delT, p.Phe32fs) in 3 of 617 patients with familial breast cancer who were sequenced. The c.96delT mutation was then detected in 29 of 12,617 unselected breast cancer cases (0.23%) compared to 11 of 4599 cancer-free women (0.24%) (OR = 0.96; 95% CI 0.48–1.93). The mutation frequency in 1988 women with familial breast cancer was 0.2% (OR = 0.84, 95% CI 0.27–2.65). Breast cancers in *XRCC2* mutation carriers and non-carriers were similar with respect to age of diagnosis and clinical characteristics. Loss of the wild-type *XRCC2* allele was observed only in one of the eight breast cancers from patients who carried the *XRCC2* mutation. No cancer type was more common in first- or second-degree relatives of *XRCC2* mutation carriers than in relatives of the non-carriers.

**Conclusion:**

*XRCC2* c.96delT is a protein-truncating founder variant in Poland. There is no evidence that this mutation predisposes to breast cancer (and other cancers). It is premature to consider *XRCC2* as a breast cancer-predisposing gene.

## Introduction

Since the discovery of *BRCA1* and *BRCA2*, more than 20 additional genes have been associated with a susceptibility to breast or ovarian cancer [1–8]. The *XRCC2* gene is a member of the *RAD51* gene family, which encodes proteins involved in homologous recombination repair of damaged DNA [9]. The *XRCC2* gene acts in the Fanconi anemia—BRCA pathway of DNA repair [10–12]. In 2012, Park et al. identified six pathogenic coding variants in 1308 women with early-onset breast cancer from Europe, North America, and Australia and no variant in 1120 controls [13]. They also described ten breast-cancer families with protein-truncating or probably deleterious rare missense variants in *XRCC2* among 689 multiple-case families [11]. Based on this, the *XRCC2* gene has been included in several clinical cancer genetic test panels [3, 14, 15].

To verify whether a *XRCC2* mutation confers elevated breast cancer risk and should be included in breast cancer test panels, we studied a large series of approximately 13,000 women with breast cancer and 5000 controls from Poland. In addition, we analyzed clinical characteristics of breast cancers in carriers of a *XRCC2* mutation, and performed loss of heterozygosity (LOH) analysis at the *XRCC2* locus in tumors from *XRCC2* mutation-positive women.

## Materials and methods

### Hereditary breast cancer cases (case series 1)

We selected 617 unrelated probands from 617 Polish families with familial breast cancer and did exome sequencing on their germline DNA [16]. We included women with a strong family history for breast cancer. Among the 617 probands with breast cancer, there were 160 women from families with at least four women affected with breast cancer, 378 women from families with three affected, and 79 women from families with two affected (at least one had bilateral breast cancer or breast cancer below age 50). The mean number of breast cancers per family was 3.4. The mean age of breast cancer diagnosis among the 617 women was 46 years (range 28–76 years). These patients were selected from a registry of 3519 familial breast cancer cases housed at the Hereditary Cancer Center in Szczecin based on the number and age of onset of breast cancer cases among their relatives, and based on that they tested negative for a panel of 17 founder Polish mutations of *BRCA1/2*, *CHEK2*, *PALB2*, *NBN*, and *RECQL* [16–20].

### Unselected cases of breast cancer (case series 2)

Unselected cases consisted of 12,679 prospectively ascertained cases of invasive breast cancer, diagnosed from 1996 to 2012, at 18 different hospitals in Poland (mean age 54, range 18–93) [19]. Patients were unselected for age, family history, and treatment. The patient participation rate was 76.1%. Information was recorded on clinical characteristics of breast cancers through review of medical records. Family history included the number of first- and second-degree relatives with cancer. 1988 patients reported at one first- or second-degree relative with breast cancer. Survival data were obtained (status: alive or dead, the date of death) from Polish Ministry of the Interior and Administration in July 2014. The Ethics Committee of Pomeranian Medical University in Szczecin approved the study.

### Controls

The control group included 4730 cancer-free Polish women aged 18 to 94 years (mean age, 53.0 years) from Poland [20].

### Sequencing of the *XRCC2* gene

We analyzed the entire coding sequence of *XRCC2* from the exome sequencing data of 617 women with hereditary breast cancer (cases series 1) using the methodology described previously [16]. In brief, the Agilent SureSelect human exome kit (V6) was used for capturing target regions. The regions were sequenced on Illumina NextSeq 500. The mean depth of coverage was approximately × 100; 97.4% of the CCDS exons were covered at × 20 depth of coverage and higher which used for variant calling.

### Genotyping for *XRCC2* c.96delT truncating mutation

We assessed DNA samples for a recurrent truncating mutation of *XRCC2* (c.96delT) in a LightCycler Real-Time PCR 480 System (Roche Life Science, Mannheim, Germany) using a TaqMan assay (Life Technologies, Carlsbad, CA, USA)—12,617 of 12,679 breast cancer cases, and 4599 of 4730 controls were successfully genotyped. All mutations were confirmed by Sanger sequencing. Sanger sequencing was performed using a BigDye Terminator v3.1 Cycle Sequencing Kit (Thermo Fisher Scientific) and ABI prism 3100 Genetic Analyzer (Thermo Fisher Scientific).

### Loss of heterozygosity analysis

Loss of heterozygosity (LOH) analysis at the *XRCC2* locus was performed in micro-dissected tumors from eight women with *XRCC2* c.96delT mutation using methodology described previously [21] with a minor modifications: (1) DNA was isolated with QIAamp DNA FFPE Tissue Kit (QIAGEN); (2) LOH was analyzed by direct Sanger sequencing of the 104 bp DNA fragment containing the *XRCC2* mutation (forward primer 5′ tctctcttcttttataagctccttg; reverse primer 5′ ttaccatgcacaggtgaatct).

### Statistical analysis

The prevalence of the deleterious *XRCC2* allele was estimated in 12,617 breast cancer cases and 4599 cancer-free women. Odds ratios were generated from two-by-two tables. Women with breast cancer, with and without the *XRCC2* mutation, were compared for age at diagnosis, clinical features of the breast cancers, and survival. Statistical significance was assessed using Fisher exact test or Chi-squared test where appropriate. Means were compared using *t* test. To estimate the survival, we followed up women from the date of diagnosis until the date of death or July, 2014, if still alive. We compared the survival between mutation carriers and non-carriers by log-rank test.

## Results

We identified a single *XRCC2* protein-truncating mutation (c.96delT, p.Phe32fs) in 3 of 617 women with hereditary breast cancer. We did not see any other truncating *XRCC2* mutation in the 617 subjects. The c.96delT mutation was present in 29 (0.23%) of 12,617 patients and in 11 (0.24%) of 4599 controls. The OR for risk of breast cancer in women with this *XRCC2* mutation was 0.96 (95% CI 0.48–1.93). The mutation frequency in 1988 women with familial breast cancer was 0.2% (OR = 0.84, 95% CI 0.27–2.65, *p* = 0.99). The OR for breast cancer risk given the *XRCC2* mutation was 1.01 for women diagnosed under 51 years of age, and was 0.92 for those diagnosed above age of 50 (Table 1).

**Table 1:** Prevalence of *XRCC2* c.96delT recurrent mutation in 12,617 women with breast cancer, by age and family history

	Total (*n*)	*XRCC2* c.96delT positive (*n*)	Prevalence (%)	OR (95% CI)	*p* value
All cases	12,617	29	0.23%	0.96 (0.48–1.93)	0.91
Age					
≤ 40	1310	3	0.23%	0.96 (0.27–3.44)	0.95
41–50	4476	11	0.25%	1.03 (0.45–2.37)	0.95
51–60	3273	5	0.15%	0.64 (0.22–1.84)	0.56
61–70	2243	7	0.31%	1.31 (0.51–3.37)	0.76
≥ 71	1315	3	0.23%	0.95 (0.27–3.43)	0.94
Number of relatives with breast cancer					
0	9710	22	0.23%	0.95 (0.49–1.96)	0.88
1	1525	3	0.20%	0.82 (0.23–2.95)	0.76
≥ 2	463	1	0.22%	0.9 (0.12–7.01)	0.92
Number of relatives with ovarian cancer					
0	11,354	26	0.23%	0.96 (0.47–1.94)	0.90
≥ 1	344	0	–	–	0.75
Cancer-free controls	4599	11	0.24%	Ref.	Ref.

The characteristics of breast cancers in patients with and without a *XRCC2* mutation are shown in Table 2. Carriers and non-carriers were similar with respect to age at diagnosis, histology, tumor size, lymph node involvement, ER, and PR status. The frequency of bilateral tumors was the same in both groups (4.2% vs. 4.6%; *p* = 0.9).

**Table 2:** Clinical characteristics of breast cancers in carriers of the *XRCC2* c.96delT mutation and non-carriers

	*XRCC2* c.96delT positive *n* = 29	*XRCC2* c.96delT negative *n* = 12588	*p* value
Age at diagnosis (years)	54.4 (36–83)	54.1 (18–93)	0.9
Follow-up (months)	55.5 (8–131)	64.2 (1–219)	0.2
Histological features			
Ductal, grade 3	4/22 (18.2%)	2027/9618 (21.1%)	0.9
Ductal, grade 1–2	8/22 (36.4)	4094/9618 (42.6%)	0.7
Ductal, grade unknown	4/22 (18.2%)	672/9618 (7.0%)	0.1
Medullary	0/22 (0.0%)	297/9618 (3.1%)	0.8
Lobular	6/22 (27.3%)	1253/9618 (13.0%)	0.1
Tubulolobular	0/22 (0.0%)	116/9618 (1.2%)	0.6
DCIS with microinvasion	0/22 (0.0%)	308/9618 (4.4%)	0.8
Other or undefined	0/22 (0.0%)	851/9618 (8.8%)	0.3
Estrogen receptor-positive	15/22 (68.2%)	6025/8667 (69.5%)	0.9
Progesterone receptor-positive	16/22 (72.7%)	5947/8359 (71.1%)	0.9
HER2-positive	6/18 (33.3%)	1278/7282 (17.6%)	0.1
Size (cm)			
< 1	1/19 (5.3%)	917/7999 (11.5%)	0.6
1–1.9	10/19 (52.6%)	3240/7999 (40.5%)	0.4
2–4.9	6/19 (31.6%)	3506/7999 (43.8%)	0.4
≥ 5	2/19 (10.5%)	336/7999 (4.2%)	0.4
Lymph node-positive	8/20 (36.4%)	3607/8251 (43.7%)	0.4
Bilateral	1/24 (4.2%)	454/9885 (4.6%)	0.7
Vital status (deceased)	6/29 (20.7%)	2101/12445 (16.9%)	0.8

Data on survival were available for 12,474 women with breast cancer. After the mean follow-up time of 64 months, there were 6 deaths (20.7%) recorded in 29 *XRCC2* mutation carriers compared with 2101 deaths (16.8%) in 12,445 non-carriers (HR = 1.36; 95% CI 0.54–3.47; *p* = 0.45, log-rank test). The 10-year survival was 76% for the carriers compared to 75% for non-carriers.

**Fig. 1:**
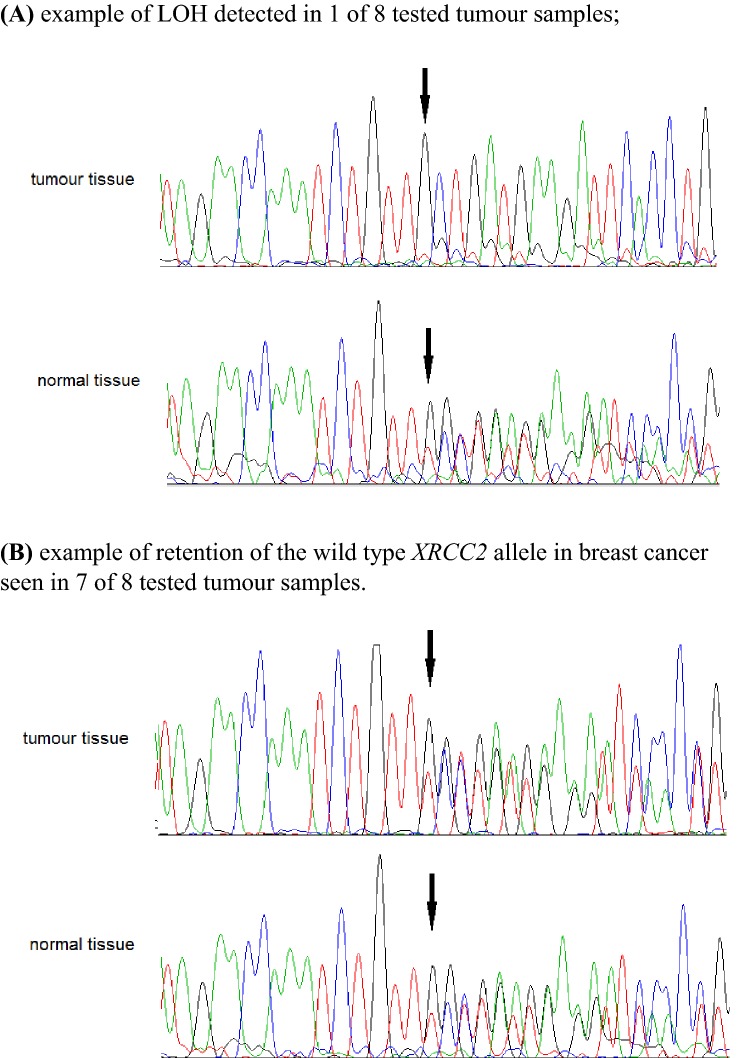
Loss of heterozygosity (LOH) analysis in breast cancer tissues from eight carriers of *XRCC2* c.96delT mutation; the c.96delT variant is indicated by arrow (↓)

Loss of the wild-type *XRCC2* allele was observed in one of the eight breast cancers from women who carried *XRCC2* c.96delT truncating mutation (Fig. 1).

To see if there might be an excess of cancer at other sites than the breast in a first- or second-degree relatives of carriers of a *XRCC2* mutation, we reviewed the pedigrees of women who have breast cancer and carry the c.96delT mutation and compared these with the pedigrees of breast cancer cases without the c.96delT mutation. No cancer type was more common in first- or second-degree relatives of *XRCC2* mutation carriers than in relatives of the non-carriers—there were in total 20 cancers in 26 families with the *XRCC2* mutation (77%) versus 8380 cancers in 11,672 *XRCC2* mutation-negative families (72%) (Table 3).

**Table 3:** Cancer family histories in first- or second-degree relatives (excluding breast cancer) reported by the 26 patients with breast cancer and the *XRCC2* c.96delT mutation compared to those reported by the 11,672 patients with breast cancer and without the *XRCC2* c.96delT mutation

Cancer site	Number (%) of cancers in relatives of *XRCC2* c.96delT positive patients (*n* = 26 families)		Number (%) of cancers in relatives of *XRCC2* c.96delT negative patients (*n* = 11672 families)	
Bladder	0	0.0%	133	1.1%
Brain	1	3.8%	251	2.1%
Colon	3	11.5%	896	7.7%
Kidney	0	0.0%	324	2.8%
Larynx	0	0.0%	452	3.9%
Lung	5	19.2%	1728	14.8%
Melanoma	0	0.0%	96	0.8%
Leukemia or Lymphoma	1	3.8%	452	3.9%
Skin (non-melanoma)	0	0.0%	147	1.3%
Pancreas	2	7.7%	336	2.9%
Prostate	2	7.7%	783	6.7%
Stomach	1	3.8%	1000	8.6%
Ovary/uterus	5	19.2%	1782	15.3%
Any site	20	76.9%	8380	71.8%

Family history in first- and second-degree relatives was available for 26 of 29 *XRCC2* mutation carriers and 11,672 of 12,588 non-carriers

## Discussion

In 2012, Park et al. suggested that *XRCC2* is associated with elevated breast cancer risk based on finding six pathogenic coding variants in 1308 women with early-onset breast cancer from Europe, North America, and Australia and no variant in 1120 controls (*p* < 0.02) [13]. We were unable to verify this association in a much larger study from a homogeneous population.

Importantly we identified a recurrent truncating mutation of the *XRCC2* gene (c.96delT, p.Phe32fs). This mutation is localized in the 5′ end of the gene at codon 32 and it is predicted to disrupt about 90% of *XRCC2* protein sequence (that includes 280 aa) including the entire Rad51 domain, and therefore it is predicted to be deleterious. However, this specific mutation appears not to be associated with an elevated risk of familial breast cancer (OR = 0.84, 95% CI 0.27–2.65) or non-familial breast cancer (OR = 0.95, 95% CI 0.49–1.96). Further, breast cancers in *XRCC2*-mutation carriers and non-carriers were similar with respect to age of onset, clinical characteristics, and survival. Loss of the wild-type *XRCC2* allele was observed only in one of the eight breast cancers from the Polish women who carried the c.96delT deletion.

Our results are similar to those of Decker et al. [22] who reported no association of *XRCC2* truncating mutations with breast cancer risk. They identified truncating *XRCC2* mutations (11 different variants, five of these predicted to affect RAD51 domain) with the same frequency (0.07%) in 9 of 13,087 breast cancer cases and in 4 of 5488 controls from the UK (OR = 0.94, 95% CI 0.26–4.19), but a twofold-increased risk could not be excluded. Hilbers et al. [23] analyzed *XRCC2* for mutations in 3548 non-*BRCA1/2* familial breast cancer cases and 1435 controls from the Netherlands, but found a protein-truncating variant in only one control. When we combine the three studies (from Poland, UK, and the Netherlands), truncating mutations of *XRCC2* were detected in 38 of 29,252 (0.13%) breast cancer cases versus 16 of 11,522 (0.14%) controls and were not associated with breast cancer risk (meta-analysis OR = 0.88, 95% CI 0.50–1.57, Mantel–Haenszel method).

It is also important to establish if missense mutations of *XRCC2* confer increased breast cancer risk. In 2016, Hilbers et al. [24] functionally characterized 27 variants in *XRCC2* by testing their ability to restore XRCC2-DNA repair-deficient phenotypes. Only the protein-truncating mutations (4 variants), but not missense variants (23 variants) were unable to restore XRCC2 deficiency. Rare non-protein-truncating variants were detected with the same frequency (0.6%) in 3548 non-*BRCA1/2* familial breast cancer cases and 1,435 controls from the Netherlands [23]. We did not detect any missense variants of *XRCC2* which are predicted to be pathogenic using in silico tools in 617 Polish families with hereditary breast cancer who were fully sequenced. These data suggest that missense variants of *XRCC2* are unlikely to be pathogenic for breast cancer.

Our study is large and population based. Poland is homogeneous from a genetic perspective and the range of mutant alleles is limited, that is reflected by the presence of a large number of founder mutations [16]. Our analysis suggests that mutations of *XRCC2* do not confer elevated breast cancer risk. Normally, a genetic counselor or physician who is given a result that a truncating mutation is present (i.e., a mutation that leads to loss of protein function) will assume that it is deleterious. In the case of the c.96delT (p.Phe32fs) mutation, our epidemiology analysis excludes this variant as pathogenic for breast cancer. It is possible that other variants (truncating or non-truncating) are pathogenic, but this will be exceedingly difficult for a counselor to prove on a single-case basis. The consequences of assigning a high-risk status to a woman based on an *XRCC2* mutation are non-trivial and may lead to increased psychological distress and possibly to unwarranted preventive surgery. In our opinion, *XRCC2* should not be included on the genetic testing panels.
